# Benzoic acid – Determination of benzoic acid in workplace air using high-performance liquid chromatography (HPLC-DAD): Air Monitoring Method – Translation of the German version from 2024

**DOI:** 10.34865/am6585e9_4or

**Published:** 2024-12-23

**Authors:** George Dragan, Ralph Hebisch, Adela Frenzen, Lutz Nitschke, Uta Lewin-Kretzschmar, Andrea Hartwig, MAK Commission

**Affiliations:** 1 Federal Institute for Occupational Safety and Health (BAuA) Friedrich-Henkel-Weg 1–25 44149 Dortmund Germany; 2 Bavarian State Office for Health and Food Safety (LGL) Pfarrstraße 3 80538 München Germany; 3 German Social Accident Insurance, Institution for the raw materials and chemical industry, Prevention - Department of Hazardous Substances, Biological Agents and Analytical Chemistry Kurfürsten-Anlage 62 69115 Heidelberg Germany; 4 Institute of Applied Biosciences. Department of Food Chemistry and Toxicology. Karlsruhe Institute of Technology (KIT) Adenauerring 20a, Building 50.41 76131 Karlsruhe Germany; 5 Permanent Senate Commission for the Investigation of Health Hazards of Chemical Compounds in the Work Area. Deutsche Forschungsgemeinschaft, Kennedyallee 40, 53175 Bonn, Germany. Further information: Permanent Senate Commission for the Investigation of Health Hazards of Chemical Compounds in the Work Area | DFG

**Keywords:** benzoic acid, air analyses, analytical method, workplace measurement, hazardous substance, high-performance liquid chromatography, diode array detection, HPLC-DAD, quartz fibre filter, silica gel

## Abstract

The working group “Air Analyses” of the German Senate Commission for the Investigation of Health Hazards of Chemical Compounds in the Work Area (MAK Commission) developed and verified the presented analytical method. This analytical method permits the determination of benzoic acid [65-85-0] occurring as inhalable particles and vapour in workplace air. The concentration range covers one tenth up to twice the currently valid occupational exposure limit value (OELV) in Germany of 0.5 mg/m^3^. Sampling is performed using a flow-regulated pump to draw a defined volume of air through a binder-free quartz fibre filter followed by a sorbent tube filled with silica gel. The volumetric flow rate is 1 l/min and the sampling duration is 2 hours (which corresponds to a sampling volume of 120 l). The collected benzoic acid is extracted with methanol and subsequently analysed by means of high-performance liquid chromatography using diode array detection. Quantitative determination is based on a multiple-point calibration function. For an air sample volume of 120 litres, the relative limit of quantification (LOQ) is 0.0023 mg/m^3^. The mean recovery is 96.3% and the expanded uncertainty for the validation range of 0.05 to 1 mg/m^3^ is 18%.

**Table:** 

**Method number**	1
**Application**	Air analysis
**Analytical principle**	High-performance liquid chromatography with diode array detection (HPLC-DAD)

## 1 Characteristics of the method

**Table:** 

**Precision:**	Standard deviation (rel.):	*s* = 1.0–1.8%
	Expanded uncertainty:	*U* = 18%
	in the range of 0.05–1 mg/m^3^ and n = 6	
**Limit of quantification:**	28 ng/ml absolute	
	0.0023 mg/m^3^ for an air sample volume of 120 l and a sampling period of 2 h	
**Recovery:**	*η* = 98–103%	
**Sampling recommendation:**	Sampling period:	2 h
	Air sample volume:	120 l
	Volumetric flow rate:	1 l/min
	for short-term measurements:	15 min; 1 l/min

## 2 Description of the substance

### Benzoic acid [65-85-0]

**Fig.1:**
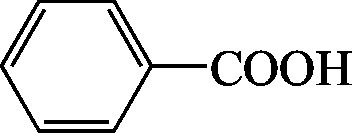
Structural formula of benzoic acid

Benzoic acid (see Figure 1) is a colourless solid that has a characteristic odour (molar mass 122.1 g/mol, melting point 122 °C, boiling point 249 °C, density 1.32 g/cm^3^). Benzoic acid is used in industry as a biocide, as a preservative in the food industry (E 210) and as a plasticiser (ECHA 2022).

Benzoic acid [65-85-0] has an occupational exposure limit value (OELV) of 0.5 mg/m^3^. It is classified in Peak Limitation Category II with an excursion factor of 4 (AGS 2023). A MAK value of 2.0 mg/m^3^ has been established for the inhalable fraction (I) and a value of 0.5 mg/m^3^ for the respirable fraction (R) (DFG 2023). In the List of MAK and BAT Values, short-term exposure to the inhalable dust has been limited by classification in Peak Limitation Category I with an excursion factor of 2 and to the respirable dust by classification in Peak Limitation Category II with an excursion factor of 4 (DFG 2023). Benzoic acid is listed in TRGS 900 (Technical Rules for Hazardous Substances) and in the List of MAK and BAT Values as a vapour/particle mixture; the substance may thus occur in the workplace air both in particle and in vapour form. A sampling device that is able to capture both the inhalable dust fraction and vapour is required for sampling (AGS 2023; DFG 2023). If the benzoic acid concentration in the inhalable dust fraction is greater than 1 mg/m^3^ or if the evaluation of the inhalable dust fraction is based on the MAK value of 2 mg/m^3^, the sample must be diluted to ensure that the analysis is performed in the validated concentration range (see Section 7).

## 3 General principles

This analytical method is used to determine the concentration of benzoic acid in the inhalable dust fraction of the workplace air in the range of one tenth to twice the OELV of 0.5 mg/m^3^ (AGS 2023).

Samples are collected by drawing a defined volume of air through a combined sampling system consisting of a binder-free quartz fibre filter and a sorbent tube (silica gel) using a suitable pump. The quartz fibre filter loaded with benzoic acid, the contents of the silica gel tube and, if applicable, the length of connecting tubing are transferred together to an amber glass vial, covered with a layer of methanol, and shaken. The sample is analysed by high-performance liquid chromatography (HPLC) with diode array detection (DAD). The quantitative evaluation is carried out using a linear calibration function.

The method is also suitable for determining benzoate concentrations in the workplace air. However, the analytical method cannot be used to distinguish between different benzoates and benzoic acid.

## 4 Equipment, chemicals and solutions

### 4.1 Equipment

For sampling:

- Sampling pump for personal or stationary sampling, suitable for a volumetric flow rate of 1 l/min (e.g. GilAir Plus, from DEHA Haan & Wittmer GmbH, 71296 Heimsheim, Germany)
- Personal sampling system for hazardous substances (PGP), inhalable dust fraction (GSP) (e.g. from GSA Messgerätebau GmbH, 40880 Ratingen, Germany)
- Filter cassette for the PGP personal sampling system for hazardous substances, plastic, with covers for 37-mm filters (e.g. from GSA Messgerätebau GmbH, 40880 Ratingen, Germany)
- Quartz fibre filters (binder-free), Ø 37 mm (e.g. MN QF-10, from Macherey-Nagel GmbH, 52355 Düren, Germany, or of comparable quality)
- Sorbent tubes packed with silica gel (e.g. Supelco ORBO 506 Activated Silica Gel (45/60) 300/150 mg, 8 × 75 mm, from Merck KGaA, 64293 Darmstadt, Germany)
- Flow meter (e.g. TSI Flowmeter 4146, from TSI GmbH, 52068 Aachen, Germany)

For sample preparation and analytical determination:

- Ultrapure water system (e.g. Millipore-Q Gradient with Elix 3UV, from Merck KGaA, 64293 Darmstadt, Germany)
- Variable piston pipettes 10–100 µl and 100–1000 µl (e.g. Reference 2, from Eppendorf SE, 22366 Hamburg, Germany)
- Bottle-top dispenser 1–10 ml (e.g. Dispensette S analog, from Brand GmbH + CO KG, 97877 Wertheim, Germany)
- Glass cutter (e.g. Supelco, from Merck KGaA, 64293 Darmstadt, Germany)
- 10-ml amber glass vials with screw caps (e.g. from CS-Chromatographie GmbH, 52379 Langerwehe, Germany)
- Laboratory compact shaker (e.g. Compact Shaker KS 14 A control, from Edmund Bühler GmbH, 72411 Bodelshausen, Germany)
- Volumetric flasks, 50 ml, glass with glass stoppers (e.g. from Brand GmbH + CO KG, 97877 Wertheim, Germany)
- Syringe filters, pore size 0.45 µm, Ø 25 mm (e.g. Chromafil RC, from Carl Roth GmbH + Co. KG, 76185 Karlsruhe, Germany)
- Disposable syringes, 5 ml, polyethylene
- Analytical balance (e.g. XPE-20S Delta Range, from Mettler-Toledo GmbH, 35396 Gießen, Germany)
- Tweezers (e.g. from Plano GmbH, 35578 Wetzlar, Germany)
- High-performance liquid chromatograph with diode array detector (e.g. HPLC 20 Nexera XR with SPD-M20A prominence diode array detector, from Shimadzu Deutschland GmbH, 47269 Duisburg, Germany)
- Autosampler (e.g. SIL-20AC XR, from Shimadzu Deutschland GmbH, 47269 Duisburg, Germany)
- C18 column, length: 250 mm; inner diameter: 2.1 mm; particle size: 5 μm (e.g. MZ-PAH 5 µm 250 × 2.1 mm, from MZ-Analysentechnik GmbH, 55129 Mainz, Germany)
- Microlitre syringe, 50 µl (e.g. from Hamilton Bonaduz AG, Bonaduz, Switzerland)

### 4.2 Chemicals

- Benzoic acid, 99% (e.g. from Merck KGaA, 64271 Darmstadt, Germany)
- Salicylic acid, 99% (e.g. from Carl Roth GmbH + Co. KG, 76185 Karlsruhe, Germany)
- Phosphoric acid, 85% (e.g. from Merck KGaA, 64271 Darmstadt, Germany)
- Methanol, ≥ 99.9% (e.g. from Merck KGaA, 64293 Darmstadt, Germany)
- Acetonitrile (ACN), HPLC ultra gradient grade (e.g. Rotisolv, from Carl Roth GmbH + Co. KG, 76185 Karlsruhe, Germany)
- Ultrapure water (ρ ≥ 18.2 MΩ × cm at 25 °C)

### 4.3 Solutions

The following solutions are prepared with the chemicals listed in Section 4.2. The solutions are stable for at least 3 months if stored in the refrigerator at + 4 ℃:

**Stock Solution 1:** (3.0 mg benzoic acid/ml in methanol)

150 mg of benzoic acid are weighed and transferred to a 50-ml volumetric flask. The flask is then filled to the mark with methanol and shaken.

The following working solutions are prepared with dilutions of Stock Solution 1:

**Working Solution 1:** 10:1 dilution of Stock Solution 1 (300 µg benzoic acid/ml in methanol)

Approx. 30 ml of methanol are placed into a 50-ml volumetric flask. 5 ml of Stock Solution 1 are added. The flask is then filled to 50 ml with methanol.

**Working Solution 2:** 10:1 dilution of Working Solution 1 (30 µg benzoic acid/ml)

Approx. 30 ml of acetonitrile (ACN) are placed into a 50-ml volumetric flask. 5 ml of Working Solution 1 are added and the flask is then filled to 50 ml with ACN.

**Working Solution 3:** 10:1 dilution of Working Solution 2 (3.0 µg benzoic acid/ml)

Approx. 30 ml of ACN are placed into a 50-ml volumetric flask. 5 ml of Working Solution 2 are added and the flask is then filled to 50 ml with ACN.

**Working Solution 4:** 10:1 dilution of Working Solution 3 (0.30 µg benzoic acid/ml)

Approx. 30 ml of ACN are placed into a 50-ml volumetric flask. 5 ml of Working Solution 3 are added and the flask is then filled to 50 ml with ACN.

### 4.4 Calibration standards

Calibration standards are prepared by diluting the working solutions with ACN in 2-ml vials according to the pipetting scheme given in Table 1. 

**Tab.1:** Pipetting scheme for the preparation of the calibration standards and the resulting concentrations

Working solution	Concentration of working solution [µg BAc/ml]	Volume of working solution [µl]	Volume of ACN [µl]	Concentration of calibration standard [µg BAc/ml]	Mass per 5 µl injection [ng BAc]
4	0.30	500	500	0.15	0.75
4	0.30	1000	0	0.30	1.5
3	3.0	200	800	0.60	3.0
3	3.0	333	667	1.0	5.0
3	3.0	467	533	1.4	7.0
3	3.0	667	333	2.0	10
3	3.0	1000	0	3.0	15
2	30	133	867	4.0	20
2	30	200	800	6.0	30
2	30	280	720	8.4	42
2	30	400	600	12	60

BAc: benzoic acid

### 4.5 Control solutions

Quality control samples are prepared as reference standards using dilutions of Stock Solution 1 and Working Solution 1 and checked regularly during the analytical run. 5 μl of the prepared sample are injected by autosampler into a high-performance liquid chromatograph and analysed under the conditions described in Section 6. The quality control samples are prepared as follows:

**Control Solution 1:** for 0.1 OELV (6.0 µg of benzoic acid in 10 ml of extraction solution or 3.0 ng per injection)

10 ml of methanol are placed into a 10-ml amber glass vial using a dispenser. A microlitre syringe is then used to spike the extraction solution with 20 µl of Working Solution 1 and the vial is shaken.

**Control Solution 2:** for 1 OELV (60 µg of benzoic acid in 10 ml of extraction solution or 30 ng per injection)

10 ml of methanol are placed into a 10-ml amber glass vial using a dispenser. A microlitre syringe is then used to spike the extraction solution with 20 µl of Stock Solution 1 and the vial is shaken.

**Control Solution 3:** for 2 OELV (120 µg of benzoic acid in 10 ml of extraction solution or 60 ng per injection)

10 ml of methanol are placed into a 10-ml amber glass vial using a dispenser. A microlitre syringe is then used to spike the extraction solution with 40 µl of Stock Solution 1 and the vial is shaken.

## 5 Sampling and sample preparation

### 5.1 Sampling

Samples are collected using stationary or personal sampling procedures. The samples taken by personal sampling are collected within the breathing zone. The inlet of the sampling head must remain unobstructed during sampling.

A binder-free quartz fibre filter is inserted into the GSP sampling head and a piece of tubing is used to attach one end of an adsorption tube (silica gel) to the GSP sampling head. The other end of the tube is connected to a pump. The adsorption tube should be attached to the GSP sampling head without a gap to avoid any loss of sample by adsorption to the tubing. If it is not possible to attach the tube to the sampling head without a gap, the tubing must be analysed as well. A flow-regulated pump is used to draw an air sample through the combined sampling system (GSP, silica gel tube) at a flow rate of 1 l/min. Over a sampling period of 2 hours, this is equivalent to an air sample volume of 120 l. A sampling record is kept of the parameters required for determining the concentration in air (air sample volume, temperature, air pressure and relative humidity). 

The flow rate must be checked for constancy after sampling. If the deviation from the adjusted flow rate is larger than ± 5%, disposal of the samples is recommended (DIN 2023 a). Tweezers are used to transfer the loaded quartz fibre filter to a 10-ml amber glass vial. The silica gel tubes are opened and the contents of the tubes (including the control layer) are transferred to 10-ml amber glass vials. If necessary, the tubing between the GSP sampling head and the silica gel tube is transferred to a 10-ml amber glass vial. 10 ml of methanol are added to all vials (filters and silica gel, if applicable the tubing). The vials are closed with an airtight seal and transported to the laboratory at room temperature.

Each series of samples should include a field blank. The only difference in the handling of this sample and the analytical samples is that no air is drawn through the filter. The field blank is stored and prepared in the same manner as the samples.

### 5.2 Sample preparation

In the laboratory, the vials containing the sampling media and, if applicable, the tubing, are shaken for at least 1 hour at 200 rpm. The extracts are filtered and aliquots are transferred to 2-ml vials. The vials are placed into an autosampler and the extracts are analysed by HPLC.

The field blank is prepared and analysed in the same manner as the collected samples.

A lab blank should also be determined.

## 6 Operating conditions

**Table:** 

**Apparatus:**	HPLC device with DAD and autosampler, e.g. HPLC 20 Nexera XR, from Shimadzu Deutschland GmbH, Germany
**Separation column:**	C18, MZ-PAH, ID 2.1 mm, L 250 mm, particle size 5 µm
**Column temperature:**	25 °C
**Detector:**	Diode array detector
**Lamp:**	D_2_
**Wavelength:**	230 nm
**Mobile phase:**	30% v/v acetonitrile, 70% v/v ultrapure water, plus 2 g phosphoric acid (85%) per litre
**Flow rate:**	0.15 ml/min
**Injection volume:**	5 µl
**Run time:**	15 min

Benzoic acid has a retention time of about 9.6 minutes under the specified conditions.

## 7 Analytical determination

After the samples are prepared as described in Section 5.2, the analytical determination is performed by injecting 5 µl of each sample into a high-performance liquid chromatograph. The samples are analysed under the conditions given in Section 6. If the resulting concentrations are above the calibration range, suitable dilutions with methanol must be prepared and the analysis carried out again. The field blank and lab blank are analysed using the same procedure as the analytical samples.

## 8 Calibration

The calibration functions are obtained by analysing the calibration standards given under Section 4.4 as described in Sections 6 and 7. The resulting peak areas are plotted against the respective concentrations. In general, the calibration function is linear in the investigated concentration range.

Control samples must be analysed every working day to verify the calibration function. A new calibration must be performed if the analytical conditions change or the results of the quality control indicate that this is necessary.

**Fig.2:**
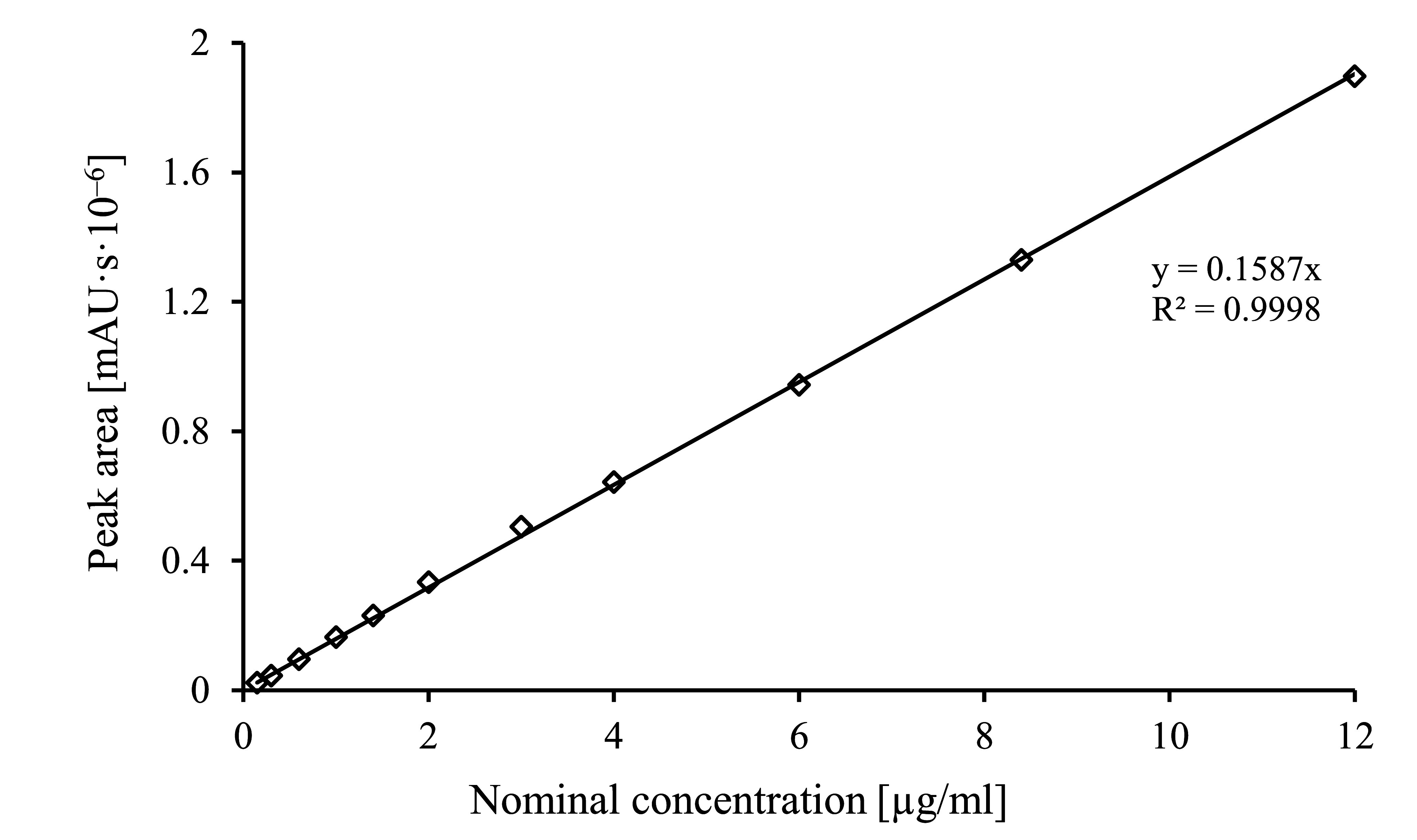
Calibration function for benzoic acid

## 9 Calculation of the analytical result

The benzoic acid concentration in the workplace air is calculated from the concentration in the measurement solution that was determined by the data analysis programme. The concentration of the investigated benzoic acid in the workplace air is calculated from the concentration in the measurement solution taking into consideration the recovery and the volume of the collected air sample by applying Equation 1 as follows:


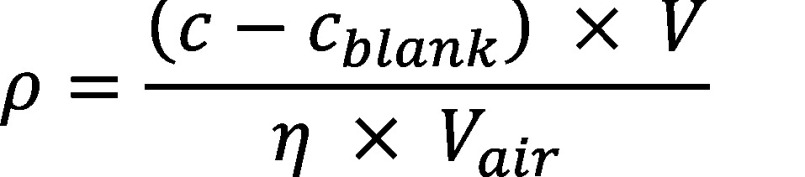
(1)

where: 

**Table:** 

*ρ*	is the mass concentration of benzoic acid in the air sample in mg/m^3^
*c*	is the concentration of benzoic acid in the measurement solution in µg/ml
*c_blank_*	is the concentration of the blank value in µg/ml
*V*	is the volume of the sample solution in l
*V_air_*	is the air sample volume in m^3^ (determined from the volumetric flow rate and the sampling time, in this case 0.12 m^3^)
*ƞ*	is the recovery

## 10 Reliability of the method

The characteristics of the method were determined according to DIN EN 482 (DIN 2021), DIN EN ISO 23861 (DIN 2023 b) and DIN 32645 (DIN 2008).

### 10.1 Precision, recovery and expanded uncertainty

The precision and expanded uncertainty were determined by spiking sets of six quartz fibre filters with different masses of benzoic acid (6 µg, 60 µg, 120 µg). One set of 6 filters was spiked with 20 µl (equivalent to 60 µg) and another set of six filters with 40 µl (equivalent to 120 µg) of Stock Solution 1 (3.0 mg/ml). Another set of 6 filters was spiked with 20 µl (equivalent to 6.0 µg) of Working Solution 1 (0.30 mg/ml). At an air sample volume of 120 l, the loads were equivalent to benzoic acid concentrations in air of 0.05 mg/m^3^, 0.5 mg/m^3^ and 1 mg/m^3^.

Each loaded quartz fibre filter was attached to a sorbent tube packed with silica gel. Air was drawn through the combined sampling system for 2 hours at a flow rate of 1 l/min. The quartz fibre filters and silica gel sorbent tubes were then prepared and analysed separately according to the steps described in Sections 5, 6 and 7.

The results were used to calculate the data for precision (see Table 2). The precision and recovery data represent the total amount of benzoic acid found on the filters and in the silica gel tubes and, where applicable, on the tubing. The distribution of benzoic acid across the filters and sampling tubes (particle and vapour phase) varied depending on the concentration tested.

**Tab.2:** Recovery, relative standard deviation and expanded uncertainty *U* for n = 6 determinations

Spiked mass [µg]	Concentration^a)^ [mg/m^3^]	Recovery [%]	Standard deviation (rel.) [%]	Expanded uncertainty ***U*** [%]
6	0.05	103.1	1.0	18
60	0.5	98.2	1.2	18
120	1	98.1	1.8	18

a) The concentration is calculated based on a 2-hour sampling period and a flow rate of 1 l/min.

The expanded uncertainty was determined by estimating all relevant influencing parameters. The two main sources of uncertainty in the measurement results are uncertainties in the sampling procedure and in the analytical procedure. 

The uncertainties contributed by the sampling procedure were estimated by determining the uncertainties associated with the air sample volume and the sampling effectiveness for inhalable dusts according to Appendix C of the standard ISO 21832 (DIN 2020).

The combined, concentration-dependent uncertainties for the entire method were calculated by combining the contributions from all sources of uncertainty. The percentages listed in Table 2 for the expanded uncertainty for the entire method were obtained by multiplying the values with the expansion factor k = 2.

### 10.2 Influence of humidity

The influence of humidity was investigated using concentrations equivalent to 0.1 and 2 times the currently valid OELV at a relative humidity of approx. 40 and 75%. The relative humidity was not found to influence the total amount of benzoic acid on the filters and sampling tubes. The deviation from the recovery (total amount of particles and vapour) was determined to be considerably below 5%.

### 10.3 Limit of quantification

The limit of quantification was determined according to DIN 32645 (DIN 2008) based on an 11-point calibration in the lower concentration range of 30 to 300 ng/ml and an injection volume of 5 µl. The absolute limit of quantification was 28 ng/ml or 0.0023 mg/m^3^ for an air sample volume of 120 litres (1 l/min over a sampling period of 2 h).

### 10.4 Capacity of the sampling system

The breakthrough behaviour of the sampling system was determined using samples spiked with benzoic acid concentrations equivalent to twice the OELV. Air with a relative humidity of approx. 75% was drawn through the sampling system for 3 hours at room temperature. No breakthrough was observed at a flow rate of 1 l/min and 3 hours of sampling. The recovery was 94.6% (total amount collected from filters and sampling tubes).

### 10.5 Storage stability

The storage stability was determined by spiking sets of six quartz fibre filters with benzoic acid concentrations equivalent to 0.1, 1 and 2 times the OELV. Air was then drawn through the sampling system for 2 hours at a flow rate of 1 l/min. The filters and the contents of the sorbent tubes were transferred to 10-ml amber glass vials and covered with a layer of 10 ml of methanol. The vials were sealed and stored for 4 weeks in the refrigerator. The extracts were then prepared and analysed as described in Sections 5, 6 and 7.

After storage for 4 weeks in the refrigerator, the mean recovery was 100.4% (0.1 OELV), 99.9% (1 OELV) and 98.8% (2 OELV) for the total amount collected from the filters and sampling tubes.

### 10.6 Selectivity

**Fig.3:**
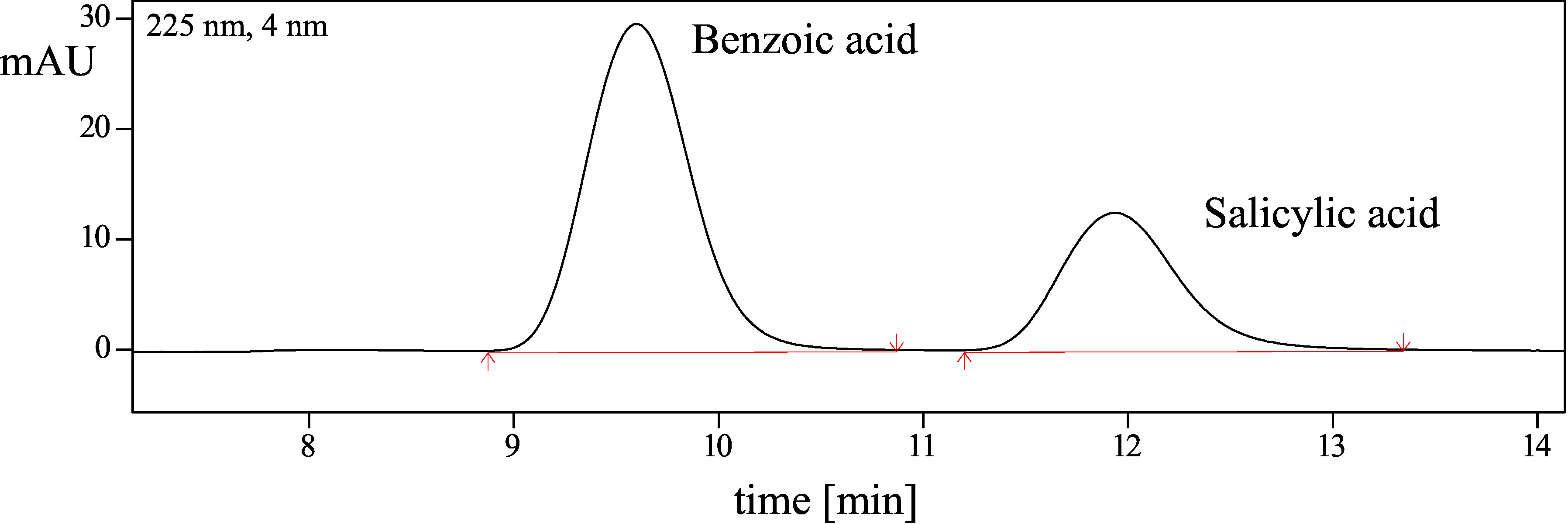
Example of a chromatogram for the separation of benzoic acid and salicylic acid by HPLC

The HPLC analytical method is specific and robust under the specified conditions. No interference was detected. It is possible to separate benzoic acid and salicylic acid by chromatography (Figure 3).

It is not possible to separate benzoic acid and other benzoates (e.g. sodium benzoate) based on the analytical results. Both substances/substance groups dissociate to form the benzoate anion in aqueous solution. Therefore, a differentiation cannot be made.

## 11 Discussion

The analytical method described above is suitable for determining benzoic acid in the workplace air in a concentration range from one tenth to twice the currently valid OELV of 0.5 mg/m^3^. The method is suitable for monitoring compliance with the short-term value. The method is also suitable for determining benzoates in the workplace air. However, the analytical method is not suitable for differentiating between benzoates and benzoic acid.

A combined sampling system for vapour/particle mixtures is required for determining benzoic acid at the workplace. The tests that were performed show that benzoic acid may evaporate from the filters during sampling. After samples were taken for 2 hours, approx. 60% of the benzoic acid that was released (at 2 OELV; 1 mg/m^3^) was recovered in the silica gel sorbent tubes.

In general, all conditions must be adapted to the HPLC device that is used, in particular those relating to sample preparation and analysis.
